# Branchio-Oto-Renal Syndrome (BOR) associated with focal glomerulosclerosis in a patient with a novel *EYA1* splice site mutation

**DOI:** 10.1186/1471-2369-14-60

**Published:** 2013-03-18

**Authors:** Maddalena Gigante, Marilena d’Altilia, Eustacchio Montemurno, Sterpeta Diella, Francesca Bruno, Giuseppe S Netti, Elena Ranieri, Giovanni Stallone, Barbara Infante, Giuseppe Grandaliano, Loreto Gesualdo

**Affiliations:** 1 Department of Medical and Surgical Sciences, University of Foggia, Foggia, Italy; 2 Department of Emergency and Organ Transplantation, University of Bari, Bari, Italy

**Keywords:** BOR syndrome, EYA1, Focal Glomerulosclerosis, Mutational analysis, RNA analysis

## Abstract

**Background:**

Branchio-oto-renal (BOR) syndrome is an autosomal dominant disorder characterized by branchial, ear, and renal anomalies. The most common gene mutated in BOR patients is *EYA1*, the human homolog of the Drosophila eyes absent gene, while mutations in *SIX1* gene, the human homolog of sine oculis, encoding a DNA binding protein interacting with EYA1, have been reported less frequently. Recently, mutations in another SIX family member, *SIX5*, have been described in BOR patients, however, this association has not been confirmed by other groups.

**Case presentation:**

In this study, we have clinically and genetically characterized a proband that displayed hearing loss, pre-auricular pits, branchial fistulae, hypoplasia of the left kidney, bilateral mild hydronephrosis, progressive proteinuria and focal glomerulosclerosis. Mutational analysis of *EYA1* gene revealed a novel splice site mutation, c.1475 + 1G > C, that affects *EYA1* splicing and produces an aberrant mRNA transcript, lacking exon 15, which is predicted to encode a truncated protein of 456 aa.

**Conclusion:**

This report provided the functional description of a novel *EYA1* splice site mutation and described for the first time a case of BOR syndrome associated with the atypical renal finding of focal glomerulosclerosis, highlighting the importance of molecular testing and detailed clinical evaluation to provide accurate diagnosis and appropriate genetic counselling.

## Background

Branchio-oto-renal (BOR) syndrome (MIM 113650) is an autosomal dominant disorder characterized by branchial, ear, and renal anomalies. This syndrome occurs with a frequency of approximately 1:40,000 in the general population and it is found in about 2% of profoundly deaf children [1]. The major clinical signs are hearing loss, branchial fistulae and pre-auricular pits, malformations of the external ear, auditory canal and mid or inner ear, and renal anomalies ranging from hypoplasia to bilateral renal agenesis [2]. Other associated clinical manifestations, although less frequent, include facial and palate anomalies, lacrimal duct aplasia and cataracts. In the absence of renal anomalies, it is defined as Branchio-oto syndrome (BO, MIM 601653) [3].

The most common gene mutated in BOR patients is *EYA1* (eyes absent homolog 1; MIM 601653) [4], the human homolog of the Drosophila eyes absent gene. Over 130 different *EYA1* disease-causing mutations, resulting in either BOR or branchial-otic syndrome (BO), have been published [5-10]. The vertebrate EYA gene family comprises four transcriptional activators that ensure normal branchial arch and epibranchial placode formation and sensory neurogenesis, including hair cell and neuron formation in the inner ear [11,12]. In the kidney, *EYA1* is a patterning gene essential for early metanephric mesenchyme development [13].

Mutations in *SIX1* gene (MIM 601295), the human homolog of sine oculis, encoding a DNA binding protein that interacts with EYA1, have also been associated with BOR syndrome although less frequently than *EYA1* mutations [14,15]. Recently, mutations in another SIX family member, *SIX5* (MIM 610896), have been reported in patients with BOR syndrome [16]. SIX5 homologous interacts with eya-1 in Caenorhabditis elegans. However, the association of *SIX5* mutations with BOR syndrome has not been confirmed by other groups and the pathogenetic role of some *SIX5* mutations was reconsider. BOR syndrome has high penetrance, but incomplete and variable expressivity. The genetic heterogeneity and the spectrum of phenotypes associated with different mutations make the diagnosis of BOR sometimes difficult. Thus, molecular analysis would be a valuable and useful tool for the confirmation of a clinical diagnosis. Here, we report the clinical and genetic diagnosis of an Italian patient with BOR syndrome associated with focal segmental glomerulosclerosis and a novel *EYA1* splice site mutation.

## Case presentation

### Patient and clinical data

**Figure 1:**
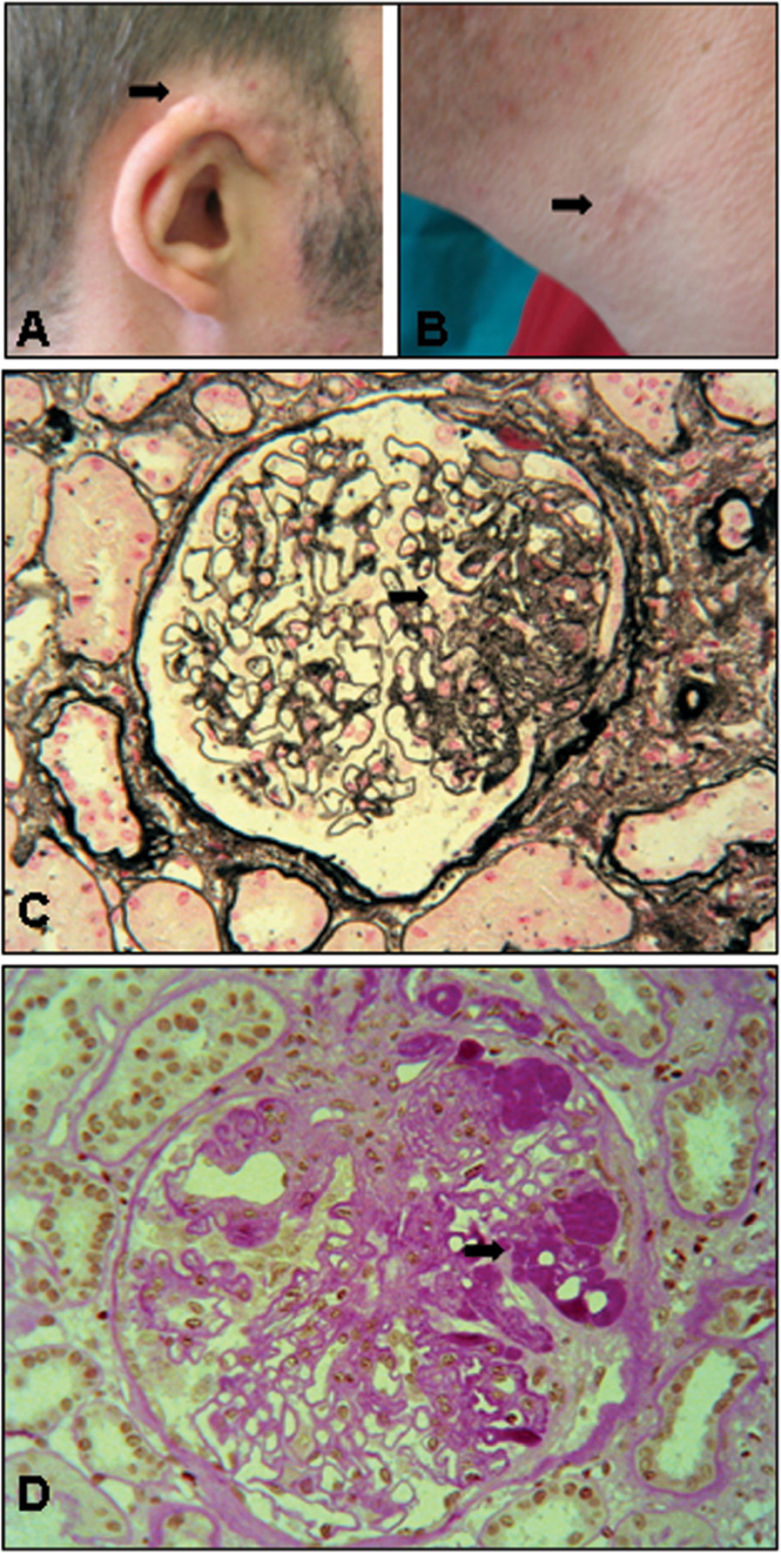
**Facial features and renal biopsy.** (**A**) Malformations of the external ear. (**B**) Surgical scars of the pre-existing fistulae. (**C**) Light microscopy on renal biopsy (Jones’ silver stain, ×400 magnification) showing focal glomerulosclerosis (*arrow*). (**D**) Light microscopy on renal biopsy (PAS stain, ×400 magnification) showing focal glomerulosclerosis (*arrow*).

A 27-year old Caucasian man, referred to our Division in November 2008, underwent to a renal biopsy because of progressive proteinuria. The patient had a history of congenital hearing impairment. At the age of 9 years, he underwent a corrective surgery of pre-auricular fistulae and lateral fistulae of the neck. At the same time, he was discovered to have a right auricular malformation, for which he had a new surgery at the age of 10 years. Urinary anomalies were first discovered at the age of 21 years. No cases of deafness, renal diseases, or branchial fistulae were reported in the patient’s family. Clinical examination showed surgical scars of the pre-existing fistulae in pre-auricular site and on both sides of the neck (Figure 1A, B). A CT scan revealed a complex dysplasia of external, middle and inner ear, with ossicular dislocation, abnormal stapedial–ovalar ratio, dysplasic vestibule and incomplete development of the cochlea of both ears. The external auditory canals were asymmetric. Audiometric exams showed a bilateral, mostly conductive hearing loss. Laboratory values showed serum creatinine of 0.9 mg/dL with a creatinine clearance of 130.6 mL/min and a proteinuria of 2.5 g/day without microhematuria. Renal ultrasound showed hypoplasia of the left kidney and bilateral mild hydronephrosis, even if the cistography did not show signs of vesicoureteral reflux. The renal biopsy was performed with histological pattern compatible with focal glomerulosclerosis (Figure 1C, D). IgM and C3 deposits were focally positive.

### Analysis of ***EYA1*** gene

This study have been performed in accordance with the Declaration of Helsinki and was approved by the Ethical Committee of University Hospital in Foggia. Informed consent for genetic studies was obtained from proband and all family members. Genomic DNA was purified from peripheral blood samples of proband and all available family members (two siblings and father), using standard procedures. Mutational analysis of *EYA1* gene (NM_000503.4; GeneID: 2138) was performed by polymerase chain reaction (PCR) and bidirectional sequencing of the coding exons and intron/exon flanking regions. *EYA1* flanking intronic primers were designed using *primer3* program (http://primer3.wi.mit.edu/). PCR products were sequenced using the Big Dye Terminator v3.1 cycle sequencing kit on 3130 Genetic Analyzer (*Life Technologies, Ltd*). *EYA1* mutation was named according to Human Genome Variation Society recommendations (http://www.hgvs.org/mutnomen). The potential effect of splice site mutation on mRNA splicing was analyzed using Splice Site Prediction server (http://www.fruitfly.org/seq_tools/splice.html). A skin biopsy was performed in order to get fibroblasts for RNA analysis. Total RNA was extracted from normal and proband’s cultured skin fibroblasts by Qiagen’s RNA Mini Kit (*Qiagen*) and cDNA was synthesized using High-Capacity cDNA Reverse Transcription Kit (*Life Technologies, Ltd*). RT-PCR and sequencing analysis were performed by specific primer pairs (F: 5^′^-CCGCTACAGACGGGTAAAAG-3^′^; R: 5^′^-CCCATACAGCAGGACTTTCG-3^′^) surrounding the region of exon 15.

**Figure 2:**
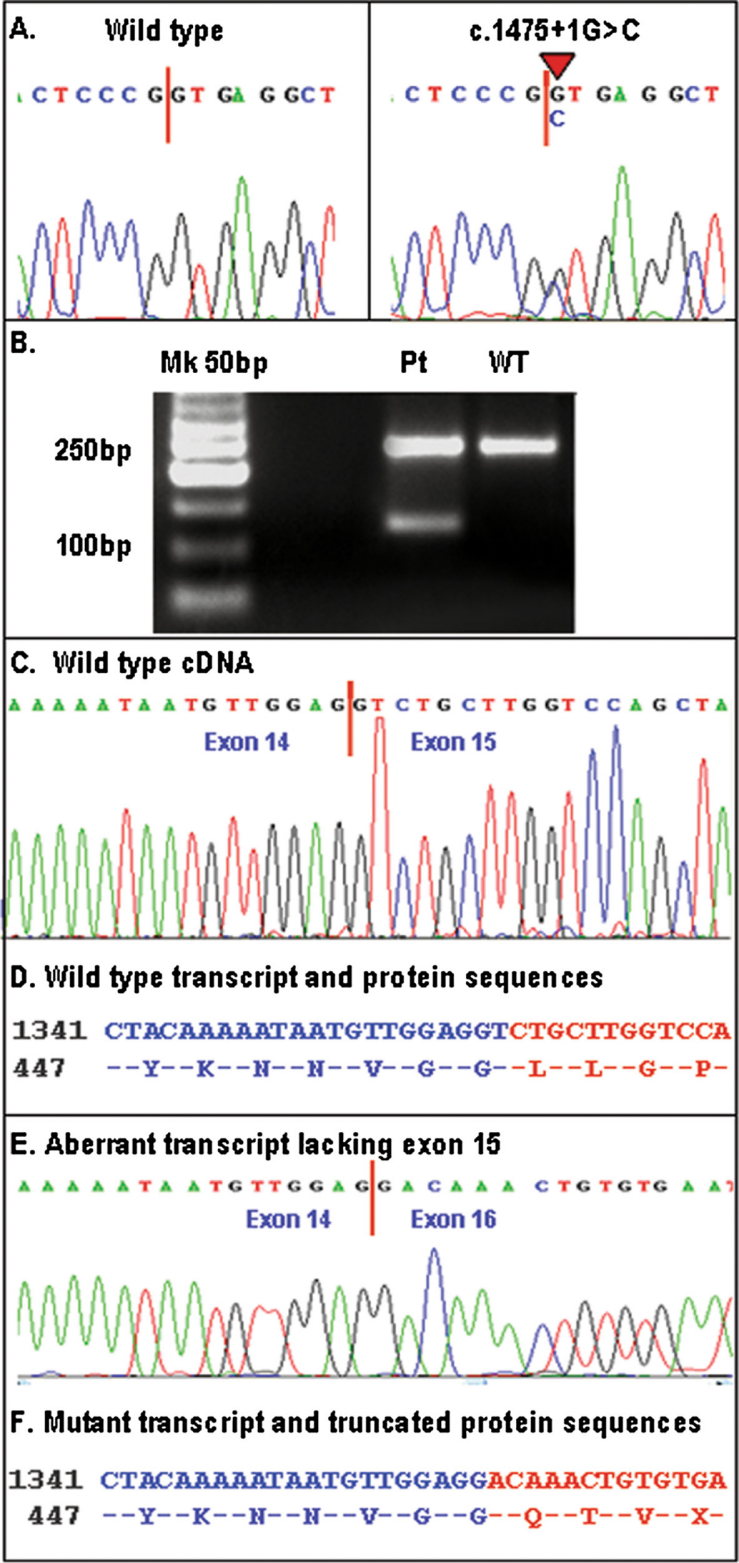
***EYA1 *mutational analysis.** (**A**) Electropherograms of wild type (left) and mutant (right) *EYA1* sequences. (**B**) RNA analysis by RT-PCR on wild type (WT) and proband fibroblats (Pt): an aberrant transcript of 121 bp was present in Pt line. (**C**) Electropherograms of wild type transcript. (**D**) Wild type cDNA and protein sequence. (**E**) Electropherograms of aberrant transcript lacking exon 15. (**F**) Mutant transcript and protein sequences.

Sequence analysis of proband revealed a novel *EYA1* heterozygous mutation in the donor splice site of exon 15, c.1475 + 1G > C (Figure 2A), which was absent in siblings, father and 100 healthy controls. Mother blood sample was not available. Splice Site Prediction server (http://www.fruitfly.org/seq_tools/splice.html) showed that this change affects heavily the predicted efficiency of the intron 15 splice donor site (score from 0.98 to 0.00). RNA was extracted from normal and proband’s cultured skin fibroblasts and cDNA was synthesized to identify *EYA1* aberrant transcripts. cDNA analysis showed that c.1475 + 1G > C mutation affects *EYA1* splicing, producing an aberrant PCR product of 119 bp lacking exon 15 (Figure 2B, E) respect to the wild type fragment of 241 bp (Figure 2B, C). The aberrant transcript is predicted to encode an EYA1 truncated protein of 456 aa (Figure 2E) compared to wild type protein of 592 aa (Figure 2D).

## Conclusions

BOR syndrome was first described in 1975 by Melnick *et al.* and it is characterized by hearing loss, renal anomalies and branchial cysts or fistulae [17]. Renal abnormalities are present in 66% of affected individuals, and about 6% of them progress to renal failure. Major renal alterations include monolateral renal agenesis, monolateral or bilateral hypodisplasia and hydronephosis caused by ureteropelvic obstruction or vesicoureteral reflux [2].

We report here a case of BOR syndrome presenting typically with bilateral conductive hearing loss, associated with a complex dysplasia of external, middle and inner ear; pre-auricular pits; branchial fistulae; hypoplasia of the left kidney; bilateral mild hydronephrosis and progressive proteinuria associated with atypical renal histological pattern compatible with focal glomerulosclerosis (Figure 1). However, in the absence of overt nephrotic syndrome, we can not exclude a form of secondary focal glomerulosclerosis, due to obstructive uropathy related to the presence of left renal hypoplasia. Mutational analysis of *EYA1* gene revealed a novel *EYA1* splice site mutation, c.1475 + 1G > C, in the donor site of exon 15. RNA analysis on skin biopsy sample showed that this mutation affects *EYA1* splicing, producing an aberrant mRNA transcript, lacking exon 15, that is predicted to encode an EYA1 truncated protein of 456 aa respect to the wild type protein of 592 aa (Figure 2). The transcriptional effects of *EYA1* mutations are often unknown due to the difficulty of obtaining appropriate samples for RNA analysis. To date, *EYA1* transcript analysis have been reported only for five other patients with BOR syndrome, two of which presented unstable *EYA1* transcripts [8] and three aberrant transcripts [15,18]. Clinical features of the latter three patients included renal hypoplasia, similar to our patient with the c.1475 + 1G > C mutation, however focal glomerulosclerosis associated with IgM and C3 positive immunofluorescence was never been reported.

EYA1 is a dual-function transcription factor, with an amino terminal transcriptional co-activator region that interacts with SIX1 and DACH, and a highly conserved 271 aa carboxy terminal Eya Domain (ED) that dephosphorylates SIX1–DACH complexes to switch from repression [14]. *In vivo* analysis showed that ED mutations impaired the catalytic activity (i.e., dephosphorylation) of the EYA1 protein, suggesting that the loss of phosphatase activity may contribute to impaired EYA1 activity and in turn to BOR phenotype [19].

The c.1475 + 1G > C mutation produces an aberrant mRNA transcript that predicts a truncated version of EYA1 protein containing only 135 aa of the conserved carboxyterminal Eya domain. The predicted protein would retain the ability to interact with SIX1–DACH, but would be unable to dephosphorylate such complexes to allow gene activation [14]. In agreement with other reports [8,18], our findings confirm that some mutant EYA1 proteins, lacking or with a disrupted phosphatase domain, might have a dominant-negative gain-of-function activity, suggesting another possible model for the pathogenesis of BOR syndrome, in addition to the previously reported haploinsufficiency model. BOR syndrome is often misdiagnosed or not diagnosed in the presence of mild clinical symptoms [20]. The hearing of BOR children with malformations of the inner ear can be exacerbated by minor environment and playground injuries (i.e., head trauma), as well as nutrition and nephrotoxic drugs might contribute to kidney failure in patients with kidney malformations. Our case is an example of a late diagnosis (27 years) of BOR syndrome, characterized by bilateral conductive hearing loss, bilateral mild hydronephrosis and progressive proteinuria associated with focal glomerulosclerosis. An accurate medical history associated with clinical, instrumental and genetic analyses lead us to diagnose BOR syndrome, even though a more timely clinical and molecular diagnosis would allow to implement nutritional and lifestyle strategies that would have prevented the most severe effects of BOR syndrome. This case suggests the opportunity of timely researching mutations of *EYA1* gene in patient affected by deafness associated with urinary anomalies and/or branchial cysts or fistulae.

## Consent

Written informed consent was obtained from the patient for publication of this case report. A copy of the written consent is available for review by the Editor-in-Chief of this journal.

## Abbreviations

CT: Computerised tomography; PCR: Polymerase chain reaction

## Competing interests

The authors declare that they have no competing interests.

## Authors’ contributions

MG carried out molecular genetic studies, analyzed data and drafted the manuscript. Md’A participated in clinical evaluation and drafted the manuscript. EM and SD participated in molecular studies. FB, GSN, GS participated in clinical evaluation; BI participated in the design of the study; ER and GG helped to draft the manuscript; LG participated in design and coordination of study and gave the final approval. All authors read and approved the final manuscript.

## Authors’ information

MG: PhD, Post-graduate school in Medical Genetics, permanent position as Biologist, University of Foggia; Md’A and FB: post-graduate school in Nephrology, University of Foggia; EM: Biologist, University of Bari; SD: laboratory technician, University of Foggia; BI, GSN and GS: MD, Nephrology, University of Foggia; ER: Associate Professor of Clinical Pathology, University of Foggia; GG: Associate Professor of Nephrology, University of Foggia; LG: Full Professor of Nephrology, University of Bari.

## Pre-publication history

The pre-publication history for this paper can be accessed here:

http://www.biomedcentral.com/1471-2369/14/60/prepub
